# Effect of comprehensive nursing intervention on quality of life and treatment outcomes in elderly patients with Gout and Hyperuricemia complicated with hypertension

**DOI:** 10.12669/pjms.40.3.7535

**Published:** 2024

**Authors:** Jing Liu, Na Lu, Shilu Shen, Wen Zhang

**Affiliations:** 1 Jing Liu, Department of Rheumatology and Immunology. Baoding No.1 Central Hospital, Baoding 071000, Hebei, China; 2 Na Lu, Department of Internal Medicine-Neurology. Baoding No.1 Central Hospital, Baoding 071000, Hebei, China; 3 Shilu Shen, Department of Rheumatology and Immunology. Baoding No.1 Central Hospital, Baoding 071000, Hebei, China; 4 Wen Zhang, Department of Rheumatology and Immunology. Baoding No.1 Central Hospital, Baoding 071000, Hebei, China

**Keywords:** Comprehensive nursing intervention, Elderly, Gout and hyperuricemia, Hypertensive complications, Treatment, Outcomes

## Abstract

**Objective:**

To evaluate the effect of comprehensive nursing intervention on the quality of life and treatment outcomes of elderly patients with gout and hyperuricemia complicated with hypertension.

**Methods:**

This is a clinical comparative study. One hundred and twenty elderly patients with gout and hyperuricemia complicated with hypertension who were hospitalized at Baoding No.1 Central Hospital were included from March 20, 2021 to March 20, 2022, and randomly divided into two groups. Patients in the control group were given conventional nursing care in the perioperative period, while those in the experimental group were given comprehensive nursing intervention on the basis of the control group. The differences between the two groups in terms of clinical effects, quality of life were compared and analyzed.

**Results:**

The response rate of the experimental group was higher than control group,with a statistically significant difference. After the intervention, the above indicators improved significantly in the experimental group compared to the control group, with statistically significant differences. There was no significant difference in Self-Rating Anxiety Scale(SAS) and Self-rating depression scale(SDS) levels between the two groups before the intervention. After the intervention, SAS and SDS decreased significantly in the experimental group compared to the control group, with statistically significant differences . The patient satisfaction rate of the experimental group was also higher than control group, with a statistically significant difference.

**Conclusion:**

Comprehensive nursing intervention is an effective treatment option for elderly patients with gout and hyperuricemia complicated with hypertension, resulting in obvious improvements in patient quality of life, improved mood, patient satisfaction and treatment outcomes.

## INTRODUCTION

Recent years have witnessed a spurt of economic development and the improvement of people’s living standards, leading to an increasingly serious phenomenon of population aging^1^, and changes in the physiological and biochemical functions of the elderly have made them an important population of medical concern. Gout and hyperuricemia complicated with hypertension are common comorbidities in the elderly. Persistently elevated blood pressure and elevated uric acid may cause cardiac, cerebral, renal, and systemic vascular damage.

In severe cases, life-threatening complications such as stroke, myocardial infarction, and heart failure occur. Gout and hyperuricemia complicated with hypertension are the primary risk factor for cardiovascular mortality.^2^,^3^ Gout is a disease caused by disorders of purine metabolism and is characterized clinically by hyperuricemia and the resulting recurrent attacks of gouty acute arthritis.In this study, comprehensive nursing interventions, including enhancing disease cognition, assisting in diet control, reasonable exercise guidance, and psychological guidance and regulation, were added to conventional antihypertensive, uric acid control, and pain improvement in patients with gout and hyperuricemia complicated with hypertension, evaluated the effect of comprehensive nursing intervention on the quality of life and treatment outcomes of elderly patients with gout and hyperuricemia complicated with hypertension.

## METHODS

This is a clinical comparative study. One hundred and twenty elderly patients with gout and hyperuricemia complicated with hypertension who were hospitalized at Baoding No.1 Central Hospital were included from March 20, 2021 to March 20, 2022. They were randomly divided into two groups: the control group and the experimental group, with 60 cases in each group. In the experimental group, there were 35 female cases and 25 male cases, aged 65-73 years old, with an average of 69.87±4.36 years old. In the control group, there were 33 female cases and 27 male cases, aged 67-74 years old, with an average of 70.57±4.23 years old. No significant difference was observed in the comparison of general data between the two groups, which was comparable (Table-I).

**Table-I:** Comparative analysis of the general data of the experimental group and the control group (*χ̅*±S) n=60.

Indicators	Experimental group	Control group	t/ (^2^	P
Age (years old)	69.87±4.36	70.57±4.23	0.89	0.37
Female (cases %)	35 (58%)	33 (55%)	0.14	0.71
Medical history (years)	4.71±1.03	4.63±1.24	0.38	0.70
BMI (kg/m^2^)	23.55±4.71	24.21±4.60	0.78	0.44
Systolic blood pressure (mmHg)	163.87±14.36	165.09±14.22	0.47	0.64
Diastolic blood pressure (mmHg)	94.43±13.52	93.61±12.75	0.34	0.73
Uric acid (μmol/L)	468.41±22.30	463.79±21.58	1.17	0.25
Comorbidity				
Hyperlipidemia (cases %)	12 (20%)	11 (18%)	0.05	0.82
Diabetes mellitus (case %)	14 (23%)	15 (25%)	0.04	0.83
Coronary heart disease (case %)	8 (13%)	6 (10%)	0.32	0.57

P>0.05.

### Ethical Approval

The study was approved by the Institutional Ethics Committee of Baoding No.1 Central Hospital(No.:2021035; Date: May 27, 2021), and written informed consent was obtained from all participants or their families.

### Inclusion criteria:

- Patients who met the diagnostic criteria for gout^4^ and were confirmed by X-ray examination.
- Patients who were conscious, without mental disorders, and able to actively cooperate with the implementation of treatment and nursing plans.
- Patients aged >65 years.
- Patients with concurrent hypertension who met the diagnostic criteria for hypertension.^5^
- Patients with complete clinical data.
-Patients who were able to cooperate with the completion of the study and had good treatment compliance.
- Families are willing to taken to complete the treatment course.

### Exclusion criteria:

- Patients with abnormal liver and kidney function.
- Patients who have recently taken uric acid-lowering drugs.
- Patients with severe psychiatric disorders and unable to cooperate satisfactorily with the study.
- Patients with severe underlying diseases such as cerebral infarction, coronary artery disease and diabetes mellitus.
- Patients with blood uric acid <420 μmol/L.

Patients in the control group were given conventional nursing care, i.e., conventional health education, to help patients and their family members get familiar with the hospital situation as soon as possible; take medicine as prescribed by the doctor and receive relevant treatment, including basic treatment of routine antihypertensive drugs, analgesic drugs, uric acid lowering, renal function protection and so on. Patients in the experimental group were treated with a comprehensive nursing intervention model on the basis of the control group, with the following main contents.

Patients were given high-calorie, low-purine and alkaline foods while being forbidden to eat raw and cold foods to promote uric acid excretion; Medication nursing: Patients were monitored to use medication as prescribed and drink plenty of water during medication. They were also observed for rash, fever, gastrointestinal discomfort, liver damage and other adverse reactions during the treatment period, and timely symptomatic treatment was carried out.

Patients with pain were told to rest in bed as far as possible, properly elevate the affected limb and cooperate with nursing measures such as physiotherapy and warmth; Complication nursing patients were monitored for related adverse reactions and liver and kidney function during early treatment.

In addition to psychological communication with patients, nurses increased communication with family members, took the initiative to understand the psychological problems of patients and family members during treatment, made appropriate interventions whenever possible, and helped patients build confidence in treatment.

Regular lectures, public number push and one-on-one lectures were carried out to provide health education to patients and their families, including the etiology, pathogenesis and treatment of gout, to help patients better understand ventilation knowledge and build confidence in overcoming the disease. Other nursing: Patients were instructed to exercise rehabilitation, work and rest, establish a normal schedule, and ensure sleep time and sleep quality.

### Observation indicators

Clinical effect evaluation marked response significant improvement in patients’ pain symptoms, systolic and diastolic blood pressure returned to normal levels, and blood uric acid returned to normal levels; Moderate response: some reduction in patients’ pain symptoms, a decrease in systolic and diastolic blood pressure compared to the previous one, and a decrease in blood uric acid; No response: No improvement in patients’ pain symptoms, no significant decrease or increase in systolic blood pressure and diastolic blood pressure, and no decrease in blood uric acid. Overall response =(marked response + moderate response)/total number of cases x 100%. Comparative analysis of the quality of life of the two groups before and after intervention.

The Generic Quality of Life Inventory-74 (GQOLI-74) was used to assess various aspects of patients such as physical function, psychological function, social function, and physical living status. A total of 20 questions and 74 items were set in the questionnaire, each item scored 1-5 points. The higher the score, the higher the quality of life of the patients.^6^ Comparative analysis of emotional status. The Self-rating Anxiety Scale (SAS) and Self-rating Depression Scale (SDS)^7^ were employed to assess the emotional change of the two groups before and after the intervention, respectively, with lower scores indicating better emotional status. Comparative analysis of patient satisfaction. The Patient Satisfaction Questionnaire Short Form (PSQ-18)^8^ was used to compare and analyze patient satisfaction before and after the intervention, including greatly satisfied, more satisfied, satisfied, uncertain, and unsatisfied. Overall satisfaction = (greatly satisfied + more satisfied + satisfied)/total number of cases x 100%.

### Statistical analysis

All data in this study were statistically analyzed by SPSS 20.0 software, and measurement data were expressed as (*χ̅*±S). Two independent sample t-test was used for comparison between groups, paired t-test was used to analyze data within groups, and χ^2^ test was used for the comparison of rates. P<0.05 indicates a statistically significant difference.

## RESULTS

The comparative analysis of the clinical outcomes of the two groups suggested that the response rate of the experimental group was 85%, higher than that of the control group (70%), with a statistically significant difference (P=0.03) (Table-II). No statistically significant difference was observed between the two groups in physical functioning, psychological functioning, social functioning and material life status scores before intervention (P>0.05). After the intervention, the above indicators improved significantly in the experimental group compared to the control group, with statistically significant differences (P=0.00) (Table-III).

**Table-II:** Comparative analysis of the clinical outcomes of the two groups (*χ̅*±S) n=60.

Group	Marked response	Moderate response	No response	Overall response
Experimental group	33	18	9	51 (85%)
Control group	27	15	18	42 (70%)
χ^2^				4.66
P				0.03

P<0.05.

**Table-III:** Comparative analysis of quality of life scores between the two groups before and after intervention (*χ̅*±S) n=60.

Indicators		Experimental group	Control group	t	p
Physical functioning	Before intervention	47.36±6.25	47.82±6.43	0.40	0.69
	After intervention*	58.79±7.73	53.06±6.82	4.31	0.00
Psychological functioning	Before intervention	52.47±6.08	53.16±6.82	0.58	0.56
	After intervention*	57.69±6.91	55.01±7.02	3.32	0.00
Social functioning	Before intervention	62.73±8.67	62.28±8.31	0.29	0.77
	After intervention*	68.41±7.84	65.57±7.33	3.05	0.00
Material life status	Before intervention	45.87±5.63	45.32±6.02	0.52	0.61
	After intervention*	51.73±6.08	47.93±6.11	3.41	0.00

* P<0.05.

Similarily no significant difference was observed in the SAS and SDS levels of the two groups before intervention(P>0.05). After the intervention, SAS and SDS decreased significantly in the experimental group compared to the control group, with statistically significant differences (P=0.00) (Table-IV). The patient satisfaction rate of the experimental group was 92%, significantly higher than that of the control group(78%), with a statistically significant difference (P=0.04) (Table-V).

**Table-IV:** Comparative analysis of the emotional status of the two groups before and after intervention (*χ̅*±S) n=55.

Indicators		Experimental group*	Control group	t	p
SAS	Before intervention	64.72±7.76	63.89±7.60	0.59	0.56
	After intervention*	54.38±7.03	58.68±7.38	3.27	0.00
SDS	Before intervention	67.03±7.46	67.17±7.31	0.10	0.92
	After intervention*	52.74±7.18	60.43±7.50	5.74	0.00

* P<0.05.

**Table-V:** Comparison analysis of patient satisfaction between the two groups (*χ̅*±S) n=60.

Group	Greatly satisfied	More satisfied	Satisfied	Uncertain	Unsatisfied	Overall satisfaction*
Experimental group	34	11	10	4	1	55 (92%)
Control group	28	12	7	9	4	47 (78%)
χ^2^						4.18
P						0.04

* P<0.05.

## DISCUSSION

It was confirmed in our study that the efficiency of patients who received comprehensive care model intervention was 85%, and health status of those patients who received general care intervention was 70%, with a statistically significant difference (P=0.03); There was no significant difference in the scores of physical function, psychological function, social function and material life status between the two groups before the intervention (P>0.05). The above indexes in the experimental group improved significantly after the intervention compared with the control group. There was no significant difference in SAS and SDS levels between the two groups before the intervention (P>0.05), but after the intervention, SAS and SDS in the experimental group were significantly lower than those in the control group, with a statistically significant difference (P=0.00); the satisfaction rate of patients in the experimental group was 92%, and that of patients in the control group was 78%, with a statistically significant difference (P=0.00).

The experimental group reported much better than the control group, with a statistically significant difference (P=0.04). It can be seen that the integrated nursing intervention model can improve the treatment effect because it is completely integrated into the treatment, allowing patients to receive targeted interventions at every stage, which is important to improve the treatment effect. Secondly, the care model also brings good feelings to the patients and makes them feel valued, which has a positive psychological impact on them. In addition, the patients’ quality of life scores were significantly higher than those of the control group after the intervention by the comprehensive nursing intervention model. Their psychological functioning, somatic functioning, social functioning, and physical living status scores were significantly improved.

The source of uric acid in the human body is mainly exogenous from the decomposition of nucleotides in purine-rich or nucleoprotein-rich foods, which accounts for about 20% of the total uric acid in the body; endogenous from the decomposition of amino acid phosphate ribose and other small molecules ribonucleic acid in the body, which accounts for about 80% of the uric acid in the body.^9^ Therefore, this is the key to the treatment of gout to control the intake of exogenous purines, reduce the source of uric acid, and promote the excretion of uric acid from the body.^10^

It was also confirmed by Desideri et al.^11^ that there is a positive correlation between serum uric acid levels and hypertension and cardiovascular effects, especially cardiovascular risk factors may have direct deleterious effects or synergistic effects with other cardiovascular risk factors. According to Gill et al and Ndrepepa.^12^,^13^, for every 1-SD increase in serum uric acid, the risk of coronary heart disease increased by 1.19 %. This study also confirmed that elevated blood pressure mediates approximately one-third of the effect of urate on cardiovascular disease risk. Uric acid-lowering therapy has a favorable effect on systolic blood pressure.

Given that the mechanisms by which gout, as well as hyperuricemia, affects patients have not been determined, drug therapy alone may not achieve satisfactory results.^14^ Only by combining health education with dietary modification and lifestyle changes can gout attacks be effectively controlled. It was found in a related study that nursing interventions taken in the treatment of gout and hyperuricemia can play a positive role.^15^ Scientific and reasonable nursing interventions are an important part of the treatment of gout and hyperuricemia.^16^ The comprehensive nursing model is an intervention method based on a continuum of thinking and has many advantages, such as being personalized, holistic and effective.^17^,^18^

With the rapid development of modern nursing, the comprehensive nursing model is also widely used in clinical practice. The integrated nursing model is more operational and easily accepted by patients than traditional nursing, and is a more practical and perfect nursing model.^19^ The comprehensive nursing intervention model can increase the nurse-patient relationship, improve patients’ psychological and psychological satisfaction and security, and enhance their confidence in overcoming the disease so that patients can actively cooperate with medical staff to receive treatment, which is conducive to the recovery of the disease.

### Limitation

It includes a small number of samples and the study was homogeneous because only elderly patients with gout and hyperuricemia complicated with hypertension were included (since hypertension caused by hyperuricemia is secondary, there is still a considerable prevalence in young and middle-aged patients). The sample size will be further expanded in future clinical work, the follow-up time will be extended, and young and middle-aged patients will be included in the study, to make the study content perfect.

## CONCLUSION

Comprehensive nursing intervention results in benefits in the treatment of elderly patients with gout and hyperuricemia complicated with hypertension. It includes significant improvement in patients’ quality of life, emotional level, a significant increase in satisfaction, significant reduction in patient’s pain, improved treatment outcome, and speedy recovery. It is of high reference value in clinical nursing and is worth promoting in the clinical practice.

### Authors’ Contributions:

**JL** and **NL:** Carried out the studies, collection of data, drafted the manuscript, are responsible and accountable for the accuracy and integrity of the work. **SS:** Performed the statistical analysis and participated in its design.

**WZ:** Participated in acquisition, analysis, or interpretation of data and draft of the manuscript. All authors read and approved the final manuscript.
